# Integrated Care Models for Severe Mental and Physical Health Disorders: A Scoping Literature Review for the EU-MIND European Initiative

**DOI:** 10.1192/j.eurpsy.2025.1626

**Published:** 2025-08-26

**Authors:** M. Rahmati, U. Isayeva, E. Touitou-Burckard, C. Gandre, A. Kiejna, M. Manchia

**Affiliations:** 1 AMU, Marseille, France; 2 Unit of Psychiatry, Department of Medical Sciences and Public Health, University of Cagliari, Cagliari, Italy; 3 IRDES, Paris, France; 4 Department of Psychology, Faculty of Education, Psychology Research Unit for Public Health, University of Lower Silesia, Wroclaw, Poland; 5 Section of Psychiatry, Department of Medical Sciences and Public Health, University of Cagliari, Cagliari, Italy

## Abstract

**Introduction:**

The co-occurrence of severe mental disorders (SMD) and physical health issues requires integrated care models that address both effectively. While interest in this area is growing, recent syntheses remain limited, particularly regarding technological advancements and the sustainability of health systems. This scoping literature review (SLR) supports the EUropean Mental and Physical Health Initiative (EU-MIND, THCS Horizon Europe) by examining integrated care for comorbid SMD and physical health conditions.

**Objectives:**

The review aimed to identify integrated care models for SMD and physical health conditions, with a focus on technology and sustainability. Findings will inform subsequent EU-MIND phases, including a Delphi survey, financial impact assessment, and feasibility pilot studies.

**Methods:**

Following PRISMA-ScR guidelines, we conducted a comprehensive search strategy in PubMed, Embase, Cochrane Library, and gray literature from inception to November 2024. Data were analyzed using the SELFIE framework, categorizing findings by WHO health system components (service delivery, governance, workforce, financing, technologies, and information).

**Results:**

We reviewed over 75 studies on integrated care models, highlighting the value of coordinated mental and physical healthcare, particularly in community settings. Digital tools such as telemedicine and electronic patient-reported measures (ePREMs, ePROMs) enable sustainable care and efficient data-sharing. Challenges identified include workforce training gaps, inconsistent financing, and limited support for addressing social determinants of health (SDOH). The integration of roles like nurse practitioners and lay workers remains underutilized due to system barriers. Collaborative care models and self-management interventions, especially those involving primary care and mental health professionals, have proven effective in reducing depressive symptoms, improving quality of life, and lowering healthcare costs. Despite progress, challenges in fully integrating care systems persist across healthcare settings. Understanding these barriers requires exploring the perspectives of all stakeholders (patients, healthcare professionals, policymakers).

**Conclusions:**

This SLR identifies key components and gaps in integrated care for individuals with comorbid SMD and physical health conditions. It highlights the importance of flexible governance, strategic integration of digital tools, and context-specific policies. Addressing these gaps can lead to more adaptive, sustainable, and patient-centered service delivery models. These findings provide a foundation for evidence-based decisions aimed at advancing multidisciplinary, integrated care approaches across European healthcare systems to better meet the complex needs of individuals with SMD.

**Disclosure of Interest:**

None Declared

